# Comparative effects of glucose and water drinks on blood pressure and cardiac function in older subjects with and without postprandial hypotension

**DOI:** 10.14814/phy2.13341

**Published:** 2017-07-12

**Authors:** Laurence G. Trahair, Sharmalar Rajendran, Renuka Visvanathan, Matthew Chapman, Daniel Stadler, Michael Horowitz, Karen L. Jones

**Affiliations:** ^1^ Discipline of Medicine The University of Adelaide Adelaide South Australia Australia; ^2^ NHMRC Centre of Research Excellence in Translating Nutritional Science to Good Health The University of Adelaide Adelaide South Australia Australia; ^3^ Cardiology Unit The Queen Elizabeth Hospital Central Adelaide Local Health Network Woodville South South Australia Australia; ^4^ Cardiology Unit Lyell McEwin Hospital Northern Local Health Network Elizabeth Vale South Australia Australia; ^5^ Aged and Extended Care Services The Queen Elizabeth Hospital Central Adelaide Local Health Network Woodville South South Australia Australia; ^6^ Adelaide Geriatrics Training and Research with Aged Care (G‐TRAC) Centre School of Medicine The University of Adelaide Adelaide South Australia Australia

**Keywords:** Autonomic function, baroreflex, blood pressure, echocardiography, glucose, hypotension

## Abstract

Postprandial hypotension (PPH) occurs frequently and is thought to reflect an inadequate increase in cardiac output to compensate for the rise in splanchnic blood flow after a meal. Gastric distension by water attenuates the postprandial fall in blood pressure (BP). Cardiac hemodynamics (stroke volume (SV), cardiac output (CO), and global longitudinal strain (GLS)) have hitherto not been measured in PPH. We sought to determine the comparative effects of water and glucose drinks on cardiac hemodynamics in healthy older subjects and individuals with PPH. Eight healthy older subjects (age 71.0 ± 1.7 years) and eight subjects with PPH (age 75.5 ± 1.0 years) consumed a 300 mL drink of either water or 75 g glucose (including 150 mg ^13^C‐acetate) in randomized order. BP and heart rate (HR) were measured using an automatic device, SV, CO, and GLS by transthoracic echocardiography and gastric emptying by measurement of ^13^ CO_2_. In both groups, glucose decreased systolic BP (*P* < 0.001) and increased HR, SV, and CO (*P* < 0.05 for all). The fall in systolic BP was greater (*P* < 0.05), and increase in HR less (*P* < 0.05), in the PPH group, with no difference in SV or CO. Water increased systolic BP (*P* < 0.05) in subjects with PPH and, in both groups, decreased HR (*P* < 0.05) without affecting SV, CO, or GLS. In subjects with PPH, the hypotensive response to glucose and the pressor response to water were related (*R* = −0.75, *P* < 0.05). These observations indicate that, in PPH, the hypotensive response to oral glucose is associated with inadequate compensatory increases in CO and HR, whereas the pressor response to water ingestion is maintained and, possibly, exaggerated.

## Introduction

Postprandial hypotension (PPH), defined as a fall in systolic blood pressure (BP) after a meal of >20 mmHg (Jansen and Lipsitz 1995), is an important clinical disorder, associated with an increased risk of syncope, falls, and mortality (Trahair et al. 2014). PPH occurs frequently – the prevalence in healthy older individuals is ~25% (Trahair et al. 2014), in residents of aged care facilities 25–40% (Trahair et al. 2014) and in patients with autonomic dysfunction 40–100% (Trahair et al. 2014). Management of symptomatic PPH is suboptimal (Trahair et al. 2014).

The pathophysiology of PPH is poorly defined (Trahair et al. 2014) but, in the broadest sense, a postprandial fall in BP implies that compensatory cardiovascular changes following a meal are inadequate. In healthy young individuals, there are postprandial increases in cardiac output (CO) and heart rate (HR) (Sidery et al. 1991; Waaler and Eriksen 1992), as well as vasoconstriction in skeletal muscle and peripheral vascular beds (Sidery et al. 1991), so there is little, if any, change in BP despite splanchnic blood pooling (Sidery et al. 1991; Waaler and Eriksen 1992). Accordingly, in healthy young subjects, meal ingestion is associated with increases in both cardiac and muscle sympathetic activity (Hayano et al. 1990; Kearney et al. 1995), and a fall in calculated vascular resistance primarily attributable to the rise in splanchnic blood flow (Kooner et al. 1989). In contrast, in healthy older subjects, there is usually a reduction in BP after a meal (Sidery et al. 1993) and the postprandial increases in CO, stroke volume (SV), and HR may be less than in healthy young (Sidery et al. 1991), although the latter have not been a consistent observation (Grobety et al. 2015), whereas increases in splanchnic blood flow appear comparable (Trahair et al. 2012). There is also a reduction, rather than an increase, in skeletal muscle vascular resistance (Kearney et al. 1995). Similarly, in response to intraduodenal glucose infusion, systolic BP falls in healthy older subjects while there is little, if any, change in young subjects (Trahair et al. 2012). However, increases in muscle sympathetic nerve activity induced by intraduodenal glucose are comparable in healthy young and older subjects probably indicative of diminished sympathetic baroreflex sensitivity, or diminished responsiveness to sympathetic neural outflow at the neurovascular junction (van Orshoven et al. 2008). Surprisingly, the effects of meal ingestion on cardiac function in older people with PPH have hitherto not been evaluated directly. In patients with sympathetic denervation, the postprandial increase in splanchnic blood flow appears to be comparable in those with PPH to healthy subjects (Puvi‐Rajasingham et al. 1999) and, in patients with autonomic failure, the sympathetic response may be diminished (Mathias et al. 1989). Furthermore, there are no changes in blood flow to the peripheral vasculature, and attenuation of postprandial increases in CO and HR, consistent with failure of the autonomic nervous system to adjust to the shift in hemodynamics (Mathias et al. 1989; Puvi‐Rajasingham et al. 1999).

Although PPH has traditionally been considered to reflect inadequate cardiovascular compensation to meal induced splanchnic blood pooling, several gastrointestinal mechanisms are now recognized to be fundamental (Trahair et al. 2014). In particular, gastric emptying (GE), which exhibits a wide interindividual variation (Collins et al. 1983), is pivotal to the regulation of postprandial BP in healthy older subjects and type 2 diabetes. When GE is relatively more rapid (Jones et al. 1998), or glucose is infused intraduodenally at a faster rate (Trahair et al. 2012), the fall in systolic BP is greater. In “healthy” older subjects PPH is associated with more rapid GE (Trahair et al. 2015) and treatments such as acarbose, which may delay both GE as well as small intestinal carbohydrate absorption, appear to be effective in management (Gentilcore et al. 2005). In contrast to the effects of small intestinal nutrient exposure, nutrient or non‐nutrient gastric distension attenuates the postprandial fall in BP in healthy older subjects and patients with PPH and probably plays a protective role in the maintenance of postprandial BP (Gentilcore et al. 2008; Vanis et al. 2010). That the fall in BP is less following oral, when compared with an identical duodenal, glucose load, in healthy older subjects is likely to reflect the “protective” effects of gastric distension (Gentilcore et al. 2009). In addition to the paucity of information about cardiac function in PPH, no studies have evaluated both the hypotensive response to oral carbohydrate and the pressor response to water drinking in patients with PPH. Accordingly, it is not known whether these are related.

We have determined the comparative effects of drinks of water and glucose on BP, HR, and cardiac hemodynamics in healthy older subjects and individuals with PPH. We hypothesized that, in response to the glucose drink, the greater fall in BP in subjects with PPH than the control subjects would be associated with inadequate compensatory increases in HR and other cardiac parameters, whereas ingestion of the water drink would elicit comparable responses between the groups.

## Materials and Methods

### Subjects

Eight healthy “older” subjects (4 male and 4 female, age 71.0 ± 1.7 years (range: 66–79 years), body mass index (BMI) 26.7 ± 1.1 kg/m^2^ (range: 20.5–30.3 kg/m^2^)), and eight subjects with documented PPH (1 male and 7 female, age 75.5 ± 1.0 years (range: 70–79 years), BMI 26.8 ± 1.2 kg/m^2^ (range: 19.8–31.6 kg/m^2^)) were recruited through an existing database, or by advertisements placed in the local hospital campus. All subjects in the PPH cohort had demonstrated a fall in systolic BP >20 mmHg within 2 h of a standardized test meal in recent research studies involving older subjects, did not have overt symptoms related to PPH, and were considered to have “idiopathic” PPH (Trahair et al. 2014). No subject with PPH was taking medication for the management of this condition. No subject in either group had a history of gastrointestinal disease or surgery, diabetes, significant respiratory or cardiac disease, alcohol abuse or epilepsy, and healthy subjects were not known to have PPH. Four subjects with PPH were taking an angiotensin II receptor antagonist and one of these was also taking a calcium channel blocker. All medication was withheld for ≥24 h prior to each study day.

### Protocol

Subjects were studied on two occasions, separated by a minimum of 1 week. On each study day, the subject attended the Echocardiography Suite in the Cardiology Unit at The Queen Elizabeth Hospital at 0800 h after an overnight fast from solids (14 h) and liquids (12 h). Upon arrival, the subject was placed in a semirecumbent position and an intravenous (IV) cannula inserted into an antecubital vein for blood sampling. An automated cuff was placed around the upper right arm to measure BP and HR. The subject was then allowed to “rest” for 15–30 min before consuming a drink (at room temperature) containing either 75 g glucose and 150 mg ^13^C‐acetate (Cambridge Isotope Laboratories, MA), made up to 300 mL with water; or 300 mL water, within 3 min; *t* = 0 min was considered as the time of completion of the drink. The order of treatments on the two study days was randomized (random number generator) prior to the enrollment of the first subject. BP, HR, and transthoracic echocardiography measurements were obtained immediately prior to the consumption of the drink until *t* = 120 min. Breath samples for the measurement of GE, and blood samples for measurement of glucose were obtained until *t* = 180 min. At *t* = 180 min the IV cannula was removed and the subject offered a light lunch prior to leaving the laboratory. On one of the 2 days following lunch, autonomic function was evaluated using standardized cardiovascular reflex tests (Piha 1991).

The protocol was approved by the Human Research Ethics Committee of the Queen Elizabeth/Lyell McEwin/Modbury Hospitals, and each subject provided written, informed consent. All experiments were carried out in accordance with the Declaration of Helsinki.

### Blood pressure and heart rate

BP and HR were measured using an automated oscillometric BP monitor (DINAMAP ProCare 100, GE Medical Systems, Milwaukee, WI), every 3 min during the “rest” period, and between *t* = 0–120 min. Baseline BP was calculated as an average of the three measurements obtained immediately prior to consumption of the drink (i.e., *t* = −9, *t* = −6, and *t* = −3 min). PPH was defined as a sustained fall in systolic BP of ≥20 mmHg (Jansen and Lipsitz 1995; Trahair et al. 2014).

### Cardiac function and systemic vascular resistance

SV, CO, ejection fraction (EF), E/e' (a measure of diastolic filing pressure), and left ventricular systolic average global longitudinal strain (GLS) were determined by transthoracic echocardiography at *t* = −3, 30, 60, 90, and 120 min. Imaging was performed using a Vivid 7 ultrasound system (GE Vingmed, Horten, Norway) and a 2.5 MHz phased array transducer, as described (Evangelista et al. 2008). Pulse‐wave Doppler and tissue Doppler imaging was used for the measurement of left ventricular diastolic function. The methods for image acquisition and postprocessing of GLS with speckle tracking have been described (Leitman et al. 2004). Briefly, the apical three‐, two‐, and four‐chamber high frame rate grayscale acquisitions (40–80 frames/sec) were obtained. Data were analyzed by a cardiologist, blinded to the subject group and treatment, with echoPAC BT‐13 level software (GE Healthcare Technologies, Sydney, NSW, Australia). GLS data were analyzed using the Q‐analysis (2‐D strain) feature and all 18 myocardial segments were averaged to obtain the GLS %. In each subject, the sonographer who conducted the echocardiography was the same on the two visits. Systemic vascular resistance (SVR), in dys·s·cm^−5^, was calculated as: 80 * mean arterial pressure (diastolic BP + 1/3[systolic BP–diastolic BP]), divided by CO (central venous pressure was considered negligible).

### Gastric Emptying

On the study day with the glucose drink, exhaled breath samples were collected in hermetically sealed 10 mL tubes (Exetainer, Buckinghamshire, England) prior to the drink (*t* = −3 min), every 5 min for the first hour, and then every 15 min for the subsequent 2 h, for assessment of GE. The ^13^CO_2_ concentration in the breath samples was measured by an isotope ratio mass spectrometer (ABCA 20/20; Europa Scientific, Crewe, UK) with an online gas chromatographic purification system. The gastric 50% emptying time (T_50_), and GE coefficient (GEC) were calculated according to the formula by Ghoos et al. (1993).

### Blood glucose

Blood glucose (mmol/L) was determined immediately using a portable glucometer (Medisense Companion 2 meter, Medisense Inc. Waltham, MA) on venous blood samples obtained at *t* = −3, 30, 60, 90, 120, and 180 min.

### Autonomic nerve function

Autonomic nerve function was assessed using standardized cardiovascular reflex tests (Piha 1991). Parasympathetic function was evaluated by the variation (R‐R interval) of the heart rate during deep breathing and the response to standing (“30:15” ratio). Sympathetic function was assessed by the fall in systolic BP in response to standing. Each test result was scored according to age‐adjusted predefined criteria as 0 =  normal, 1 =  borderline, and 2 =  abnormal for a total maximum score of 6. A score ≥3 was considered to indicate autonomic dysfunction (Piha 1991). Orthostatic hypotension (OH) was defined as a reduction in systolic BP of >20 mmHg within 3 min of standing.

### Statistical analysis

BP, HR, SV, CO, EF, E/e', GLS, and SVR were assessed as changes from baseline, whereas GE and blood glucose were analyzed as absolute values. The maximum change from baseline (rise or fall) for BP and HR were calculated. Areas under the curve (AUCs) between *t* = 0–120 min were calculated using the trapezoidal rule. Initial rises (*t* = 0–15 min) and changes in each variable over time (*t* = 0–120 min), during each condition, were assessed with one‐way repeated measures ANOVA. Differences between the conditions (i.e., treatment x time effect) and between subject groups (i.e., treatment x group effect) were assessed with two‐way repeated measures ANOVA. Differences between the treatments and subject groups were assessed with two‐way repeated measures ANOVA of AUCs. Maximum changes from baseline, GE parameters and AUCs were compared with Student's paired *t*‐test. Relationships between the variables were assessed with Pearson's correlation. A *P* < 0.05 was considered significant in all analyses. Data are presented as mean ± SEM.

## Results

The studies were well tolerated and there were no adverse events. Baseline variables are summarized in Table**** 1 **.** Subjects with PPH were slightly older than the control subjects (*P* < 0.05). No subject had evidence of autonomic neuropathy (control: score 1.1 ± 0.2, PPH: 0.88 ± 0.2, *P* = 0.45). Two subjects with PPH, but no healthy subject, had OH. In one healthy, and one PPH subject, GE data were unavailable due to degradation of the breath samples. In one subject with PPH, capillary, rather than venous, blood glucose was collected due to restricted venous access. In one subject with PPH, analysis of GLS was not feasible, as the data could not be retrieved by the echoPAC software.

**Table 1:** Baseline variables prior to each treatment in healthy older subjects and subjects with PPH

	Healthy older subjects			PPH			*P* value (healthy older subjects vs. PPH)
	Water	Glucose	*P* value (water vs. glucose)	Water	Glucose	*P* value (water vs. glucose)	
Systolic BP (mmHg)	129.8 ± 4.8	123.1 ± 4.3	<0.05	147.8 ± 3.3	137.5 ± 6.1	0.10	<0.05
Diastolic BP (mmHg)	72.6 ± 2.5	69.3 ± 2.7	0.11	73.4 ± 1.6	69.0 ± 2.8	0.10	0.94
Heart Rate (BPM)	62.6 ± 2.8	63.4 ± 3.3	0.72	63.4 ± 2.8	62.4 ± 2.1	0.45	0.97
Stroke Volume (mL)	60.9 ± 3.7	57.3 ± 2.6	0.06	64.8 ± 2.7	63.2 ± 4.0	0.47	0.29
Cardiac Output (L)	3.8 ± 0.2	3.7 ± 0.2	0.20	3.9 ± 0.4	4.0 ± 0.4	0.44	0.59
Ejection Fraction (mL)	59.9 ± 0.9	60.4 ± 1.7	0.72	59.8 ± 1.2	59.1 ± 1.7	0.37	0.72
E/e'	9.0 ± 1.2	8.6 ± 0.8	0.49	10.7 ± 0.6	9.8 ± 0.4	0.07	0.16
Global Longitudinal Strain (%)	−15.5 ± 0.5	−16.2 ± 0.6	0.23	−17.7 ± 1.1	−17.1 ± 1.2	0.34	0.12
Systemic Vascular Resistance (Dyn s∙cm^−5^)	1949.9 ± 88.1	1918.3 ± 100.3	0.55	2144.6 ± 242.5	1931.2 ± 192.8	0.11	0.66
Blood Glucose (mmol/L)	5.3 ± 0.2	5.4 ± 0.2	0.36	5.3 ± 0.1	5.5 ± 0.1	0.20	0.77

All values are mean ± SEM. BP; blood pressure, BPM; beats per minute, PPH; postprandial hypotension.

### Systolic blood pressure

#### Healthy older subjects

**Figure 1:**
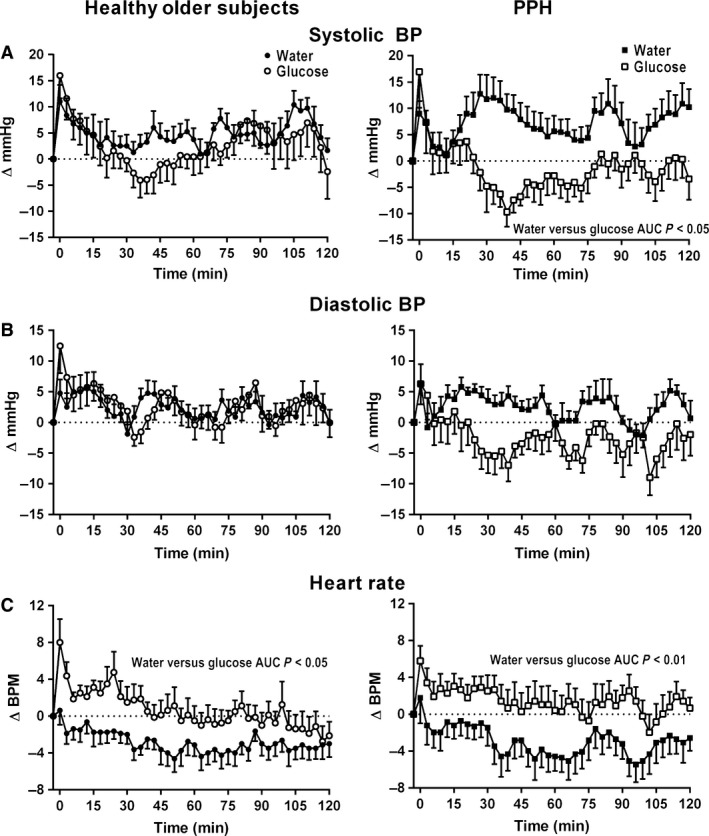
(A) Systolic blood pressure (BP), (B) diastolic BP, and (C) heart rate before and after 300 mL drinks of 75 g glucose (open symbols) and water (closed symbols) in healthy older subjects (*n* = 8, circle) and subjects with postprandial hypotension (PPH) (*n* = 8, square).

Following the glucose, but not the water drink, there was a transient rise (time effect: *P* < 0.05) in systolic BP, with a return to baseline at *t *= ~15 min (Fig. 1A). Between *t* = 0–120 min there was a modest decrease following the glucose drink (time effect: *P* < 0.001), and no overall change following the water drink (time effect: *P* = 0.68) with no difference in the AUCs for each treatment (*P* = 0.47).

#### PPH subjects

Following ingestion of both drinks, there was a transient rise (time effect: *P* < 0.01 for both) in systolic BP (Fig. 1A). After the glucose drink, there was a decrease in systolic BP (time effect: *P* < 0.001) and the AUC was less following the glucose drink (*P* < 0.05). In six of the eight PPH subjects the maximum fall in systolic BP was >20 mmHg and in the other two the fall was >10 mmHg. In contrast, following water there was a rise in systolic BP which was sustained until *t* = 120 min and systolic BP between *t* = 0–60 min was higher following the water drink (treatment x time: *P* < 0.001).

#### Comparison between the groups

The maximum fall in systolic BP following glucose was greater (treatment x group: *P* < 0.05) in the subjects with PPH, compared with the controls. There was a trend for the rise in systolic BP following water between *t* = 0–120 min to be greater (treatment x group: *P* = 0.07) in the PPH group.

### Diastolic blood pressure

#### Healthy older subjects

Following the glucose, but not the water drink, there was a transient rise (time effect: *P* < 0.05) in diastolic BP (Fig. 1B). Between *t* = 0–120 min there was a modest decrease following the glucose drink (time effect: *P* < 0.001) and no overall change following the water (time effect: *P* = 0.24), with no difference in the AUCs for each treatment (*P* = 0.87).

#### PPH subjects

Following the glucose, but not the water drink, there was a transient rise (time effect: *P* < 0.05) in diastolic BP (Fig. 1B). Between *t* = 0–120 min, there was a decrease (time effect: *P* < 0.001) after the glucose drink and no overall change following the water drink (time effect: *P* = 0.13). There was no difference in the AUCs for each treatment (*P* = 0.11).

#### Comparison between the groups

There was a trend for the maximum fall in diastolic BP following the glucose drink to be greater (treatment × group: *P* = 0.09) in subjects with PPH.

### Heart rate

#### Healthy older subjects

Following the glucose drink, there was a modest rise (time effect: *P* < 0.001), whereas after the water drink there was a modest decrease (time effect: *P* < 0.001) in HR (Fig. 1C). The AUC for HR was greater following the glucose drink (*P* < 0.05).

#### PPH subjects

Following the glucose drink there was a modest rise in HR (time effect: *P* < 0.05), with no change (time effect: *P* = 0.27) following the water drink (Fig. 1C). The AUC for HR was greater following the glucose drink (*P* < 0.01).

#### Comparison between the groups

While there was no difference in the AUCs for each treatment between the groups (treatment x group: *P* = 0.64), the maximum increase in HR after the glucose drink was greater in the healthy older subjects (treatment x group: *P* < 0.05).

### Cardiac function

#### Healthy older subjects

**Figure 2:**
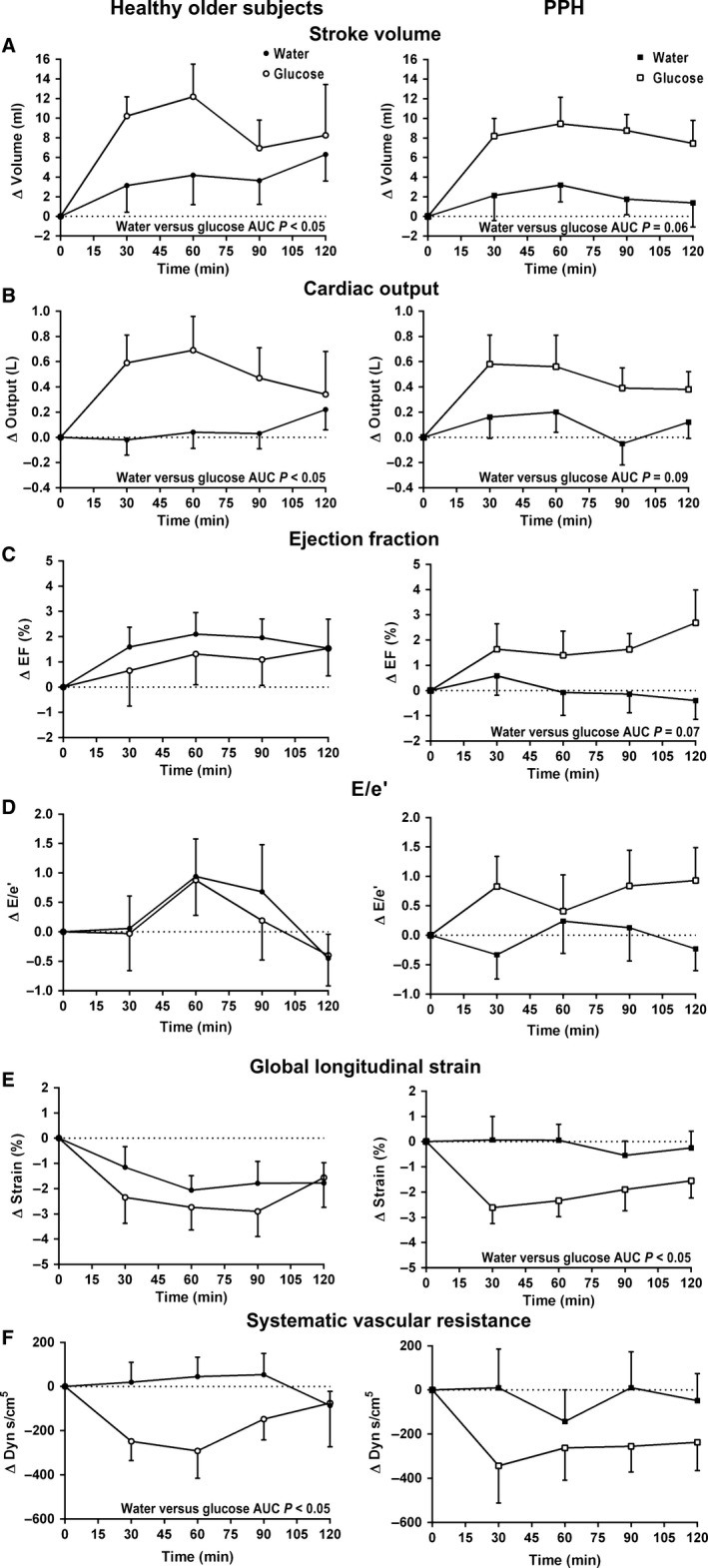
(A) Stroke volume, (B) cardiac output, (C) ejection fraction, (D) E/e', (E) global longitudinal strain, and (F) systemic vascular resistance before and after 300 mL drinks of 75 g glucose (open symbols) and water (closed symbols) in healthy older subjects (*n* = 8, circle) and subjects with postprandial hypotension (PPH) (*n* = 8, square).

Following the glucose drink, there was an increase in SV (time effect: *P* < 0.05), (Fig. 2A) and CO (time effect: *P* < 0.05) (Fig. 2B), with a trend for a decrease in GLS (time effect: *P* = 0.08) (Fig. 2E) and no change in EF (time effect: *P* = 0.65) (Fig. 2C) or E/e' (time effect: *P* = 0.15) (Fig. 2D).

Following the water drink, there was a trend for a small increase in SV (time effect: *P* = 0.08), with no change in CO (time effect: *P* = 0.34), EF (time effect: *P* = 0.10), E/e' (time effect: *P* = 0.76) or GLS (time effect: *P* = 0.14).

There was a difference between the treatments in the AUCs for SV (*P* < 0.05) and CO (*P* < 0.05).

#### PPH subjects

Following the glucose drink, there was an increase in SV (time effect: *P* < 0.001) (Fig. 2A) and CO (time effect: *P* < 0.05) (Fig. 2B), with a trend for an increase in EF (time effect: *P* = 0.10) (Fig. 2C), and a decrease in GLS (time effect: *P* < 0.01) (Fig. 2E), and no change in E/e' (time effect: *P* = 0.18) (Fig. 2D).

Following the water drink, there was no change in SV (time effect: *P* = 0.56), CO (time effect: *P* = 0.38), EF (time effect: *P* = 0.74), E/e' (time effect: *P* = 0.66), or GLS (time effect: *P* = 0.84).

There was a difference between the treatments in the AUCs for GLS (*P* < 0.05), with a trend for a difference for SV (*P* = 0.06), CO (*P* = 0.09), and EF (*P* = 0.07).

#### Comparison between the groups

There were no differences between the groups in either EF, SV, CO, or E/e' after either drink. For GLS, there was a treatment effect between the groups (treatment effect: *P* < 0.05), so that GLS was lower following the glucose drink, irrespective of subject group.

### Systemic vascular resistance

#### Healthy older subjects

Mean SVR was initially lower following the glucose drink, but this change was not significant (time effect: *P* = 0.17), and there was no change following the water drink (time effect: *P* = 0.48). There was a difference in the AUC between the treatments (*P* < 0.05) (Fig. 2F).

#### PPH subjects

Mean SVR exhibited a sustained fall following the glucose drink (time effect: *P* = 0.06), and there was no change following water (time effect: *P* = 0.74). There was no difference in the AUCs between the treatments (*P* = 0.16) (Fig. 2F).

#### Comparison between the groups

There was a treatment effect between the groups (*P* < 0.05), so that SVR was less following the glucose drink, irrespective of subject group, but there were no group (*P* = 0.63) or treatment x group effects (*P* = 0.96).

### Blood glucose

#### Healthy older subjects

There was a sustained increase in blood glucose following glucose (time effect: *P* < 0.001), and no change following water (time effect: *P* = 0.42), with a difference in the AUCs between the treatments (*P* < 0.001).

#### PPH subjects

There was a sustained increase in blood glucose following glucose (time effect: *P* < 0.001) and no change in blood glucose following water (time effect: *P* = 0.75), with a difference in the AUCs between the treatments (*P* < 0.005).

#### Comparison between the groups

There was no difference in AUC for each treatment, between the two groups (*P* = 0.62).

### Gastric emptying

There was no difference in GE of glucose between the groups (Control T_50_ 199 ± 32 min, PPH T_50_ 282 ± 37 min, *P* = 0.68; Control GEC 3.47 ± 0.12, PPH GEC 3.50 ± 0.11, *P* = 0.88).

### Relationships between responses to water and glucose

In the PPH group, but not the controls, the initial (*t* = 15–20 min) response to ingestion of water and glucose were related, for example, at *t* = 18 min, (*R* = 0.83, *P* < 0.05). There was also an inverse relationship between the fall in systolic BP with glucose and the rise during water at *t* = 45 min (*R* = −0.75, *P* < 0.05).

## Discussion

This study evaluated the comparative effects of 300 mL 75 g glucose and water drinks on BP, HR, and cardiac hemodynamics in healthy older subjects and patients with PPH. In both groups, oral glucose was associated with a fall in BP, increases in HR, SV, CO, and improvement in GLS, whereas ingestion of water was associated with an increase in BP and a modest reduction in HR, without changes in either SV, CO, EF, or GLS. In patients with PPH, the pressor response to water tended to be increased and more sustained. The falls in systolic and diastolic BP in response to glucose were, predictably, substantially greater in the PPH group, but the compensatory increase in HR was comparable in both groups. Interestingly, in the PPH group, there was an inverse relationship between the hypotensive response to glucose and the hypertensive response to water. These observations accordingly suggest that, in PPH, the hypotensive response to oral glucose is associated with inadequate compensatory increases in HR and CO, whereas the pressor response to water ingestion is maintained and, possibly, exaggerated.

Our study is the first to evaluate cardiac function directly (i.e., SV, CO, EF, and GLS) in older subjects with PPH. The groups were reasonably well matched demographically, that systolic BP at baseline was higher in the PPH than control group is not surprising given that hypertension is known to predispose to PPH (Trahair et al. 2014). A 75 g glucose load has been used widely in the diagnosis of PPH (Trahair et al. 2014). That the fall in BP in response to oral glucose was more marked for systolic than diastolic BP, was to be expected, given that the former is primarily dependent on preload and contractility, both of which would be reduced with inadequate increase sympathetic output, whereas the latter is dependent on peripheral vascular resistance (Guyenet 2006). It is appreciated that there is some variability in the postprandial hypotensive response (Trahair et al. 2014), however, in six of eight PPH subjects the maximum fall in systolic BP was >20 mmHg, and in the two others the latter was >10 mmHg. Hence, the hypotensive response to glucose did differ markedly between the two groups. The rate of GE, which is a determinant (Jones et al. 1998), as well as a risk factor (Trahair et al. 2015), for PPH was comparable in the two groups; this lack of difference is likely to be attributable to the modest size of the two groups. Our PPH subjects were “asymptomatic” without overt conditions likely to have affected autonomic function and it is not surprising that none had abnormal cardiovascular autonomic function. There were consistent cardiac hemodynamic responses to glucose with increases in SV and CO and an improvement in GLS; the latter is a robust marker of global left ventricular function and diminished GLS is recognized as a predictor of adverse cardiac events (Kalam et al. 2014). These responses were not influenced by PPH. In healthy young and older subjects intraduodenal glucose infusion is known to be associated with comparable increases in mesenteric blood flow and vascular conductance (Trahair et al. 2012) Taken together, our observations, accordingly, indicate that in PPH the hypotensive response to oral glucose is associated with inadequate compensatory increases in both baroreceptor and myocardial function greater than that which is associated with normal aging (Gribbin et al. 1971). An unresolved, and important, issue relates to the initial insult that is responsible for inadequate cardiac compensation to oral glucose. If diversion of blood to the splanchnic circulation represents the primary problem it may be anticipated that there would be a consequent reduction in SV, which was not the case – SV increased substantially following glucose ingestion. An alternative explanation is that a reduction in SVR leads to a reflex increase in sympathetic tone with an increase in HR, potentially splanchnic venoconstriction, and consequent increases in venous return and SV, both resulting in an increase in CO. However, there was no significant difference in the mean fall in SVR between the two groups.

Subjects with PPH exhibited a substantial pressor response to the water drink, which tended to be greater, and more sustained, than in health. The response also appeared biphasic, with a nadir at ~15 min followed by a subsequent, sustained rise. This elevation in blood pressure was not associated with changes in SV, CO, EF, GLS, or SVR, but a modest reduction in HR, as has been reported in patients with autonomic neuropathy (Jordan et al. 2000; Cariga and Mathias 2001). It has been suggested that impairment of baroreflex function accounts for the pressor response to water in healthy older, but not young, subjects, as attested to by the modest fall in HR in comparison to the substantial rise in BP (Jordan et al. 2000). This concept is supported strongly by the outcome of studies in animals (McHugh et al. 2010). Gastric distension induced by a balloon increases muscle sympathetic nerve activity, the so‐called “gastrovascular reflex,” a response known to be attenuated in the healthy elderly (van Orshoven et al. 2004). Water drinking also increases peripheral resistance (Cariga and Mathias 2001) and may attenuate orthostatic tachycardia in patients with idiopathic orthostatic intolerance (Shannon et al. 2002). Jordan et al. (2000) reported that the pressor response to water was exaggerated in patients with autonomic failure, some of whom had PPH (consistent with our observations) despite reduced release of noradrenaline, and postulated that this may reflect upregulation of vascular α_1_‐adrenoreceptors and/or impaired baroreflex buffering (Jordan et al. 2000). While direct stimulation of sympathetic activity triggered by visceral stretch is likely to be important, as evidenced by the response to distension of the stomach with a balloon (van Orshoven et al. 2004), changes in intravascular volume may also be relevant (Cariga and Mathias 2001). There is also evidence that hypo‐osmotic signaling via hepatic afferent fibers may influence sympathetic outflow directly via a local spinal reflex (May et al. 2011). Girona et al. (2014) reported that the effect of water ingestion on cardiac function in healthy young individuals is also dependent on its temperature; ingestion of water at 30°C and 22°C increased SV, whereas water at 37°C did not (Girona et al. 2014). Recently, Grobety et al. (2015) studied healthy older adults who drank either 100 mL or 500 mL of water before breakfast and reported that the postprandial fall in systolic BP was less in response to 500 mL, which tended to increase SV and CO more (Grobety et al. 2015). Our observations add to the recommendation for the use of water drinking in the management of PPH (Trahair et al. 2014).

In interpreting our observations, some limitations should be recognized. In particular, the number of subjects with PPH was small, and none had severe PPH or PPH associated with autonomic dysfunction as assessed by cardiovascular reflex testing. Moreover, the methodology used to evaluate autonomic dysfunction was less than optimal. The control group was not well matched for age or gender and baseline systolic BP was higher in the PPH group which, as discussed, would favor a greater fall in BP. Cardiac parameters, BP and HR were, of necessity, evaluated intermittently, rather than on a continuous basis, albeit, relatively frequently and using 2‐D, rather than 3‐D imaging. We did not measure splanchnic blood flow, or vascular responses in different beds (e.g., skeletal muscle), so our observations regarding the pathophysiology of PPH are limited to cardiovascular hemodynamics. Blood glucose was measured using a glucometer, with inherent limitations in precision, but the observed rise after the glucose drink was substantial.

In conclusion, in PPH, the hypotensive response to oral glucose is associated with inadequate cardiac compensation, the acute pressor response to water ingestion is sustained and, possibly, more pronounced and hypotensive and pressor responses are inversely related.

## Conflict of Interest

MH has participated in Advisory Boards and/or symposia for Novo Nordisk, Sanofi, Novartis, Eli Lilly, Merck Sharp & Dohme, Boehringer Ingelheim, and AstraZeneca and has received honoraria for this activity. None of the other authors have any personal or financial conflict of interest to declare.
